# Relating Physical Activity to Cognitive Function, Brain Morphology, and AD‐related Blood Biomarkers: Preliminary Findings from the IGNITE study

**DOI:** 10.1002/alz70855_102756

**Published:** 2025-12-23

**Authors:** Lauren Oberlin, Audrey M. Collins, Eric D Vidoni, Emma Tinney, Hayley Ripperger, Lu Wan, Lauren Raine, Cristina Molina‐Hidalgo, George Grove, Haiqing Huang, Patricio Solis‐Urra, Kelsey R. Sewell, Thomas K Karikari, Jill K Morris, Chaeryon Kang, Anna Marsland, Arthur F. Kramer, Charles Hillman, Jeffrey M. Burns, Edward McAuley, John Jakicic, Kirk I. Erickson

**Affiliations:** ^1^ AdventHealth Neuroscience Institute, Orlando, FL, USA; ^2^ Weill Cornell Medicine, New York, NY, USA; ^3^ AdventHealth Research Institute, Orlando, FL, USA; ^4^ University of Kansas Medical Center, Alzheimer's Disease Research Center, Fairway, KS, USA; ^5^ Northeastern University, Boston, MA, USA; ^6^ University of Pittsburgh, Pittsburgh, PA, USA; ^7^ AdventHealth Research Institute, Neuroscience, Orlando, FL, USA; ^8^ Advent Health, Orlando, FL, USA; ^9^ Center for Cognitive & Brain Health, Northeastern University, Boston, MA, USA; ^10^ University of Kansas Alzheimer's Disease Research Center, Kansas City, KS, USA; ^11^ University of Illinois, Urbana, IL, USA; ^12^ University of Kansas Medical Center, Kansas City, KS, USA

## Abstract

**Background:**

Physical activity (PA) may be a potent strategy to preserve cognitive function and reduce dementia risk, though critical questions regarding effectiveness, dosing, and mechanisms remain. Identifying the clinical impact and biological pathways of PA will help direct new preventive approaches and improve the precision of behavioral interventions. We present preliminary cross‐sectional and longitudinal findings from a phase 3 clinical trial of aerobic exercise on cognition and brain health in older adulthood.

**Methods:**

The *Investigating Gains in Neurocognition in an Intervention Trial of Exercise(*IGNITE) study is a 12‐month randomized dose‐response exercise trial in 648 older adults (69.88 ± 3.75 years [range 65‐80]; 71% female; 25% non‐white) consisting of three conditions: (1) 150 minutes/week moderate‐intensity aerobic exercise, (2) 225 minutes/week moderate‐intensity aerobic exercise, or (3) 150 minutes/week light‐intensity stretching/toning (control). We obtained a comprehensive cognitive assessment, brain MRI, PET amyloid, Alzheimer's Disease(AD)‐related blood biomarkers (ptau217, GFAP, NfL, Aβ1‐42/Aβ1‐40), accelerometry and cardiorespiratory fitness (CRF) data at baseline and 12‐months.

**Results:**

Higher baseline fitness (CRF) was associated with greater volume of select hippocampal subfields, preserved white matter microstructural integrity, and better performance across multiple cognitive domains including executive function, processing speed, and episodic memory. Greater baseline moderate‐to‐vigorous PA (MVPA) was associated with better multi‐domain cognitive performance and decelerated brain aging. Higher fitness and MVPA were also related to lower NfL levels, particularly among Aβ‐positive individuals, suggesting that maintaining higher fitness may help offset neurodegenerative changes in the setting of preclinical AD pathology. In longitudinal analysis, total adverse event risk was low, emphasizing general safety of aerobic exercise in this large community‐based sample. During this talk, we will also share preliminary interventional findings on the impact of exercise on executive function and other cognitive processes, dose‐response relationships, and specific demographic, health, and genetic factors that may influence the potency of exercise effects on cognitive outcomes.

**Conclusions:**

Preliminary findings emphasize the relationship of fitness and PA to cognition, multiple aspects of brain morphology, and AD‐related blood biomarkers in cognitively unimpaired older adults. This research also identifies multiple moderators of these relationships, which may help inform individualized exercise prescriptions to optimize cognitive and brain health in older adulthood.

